# Cronic non-bacterial osteomyelitis (CNO) in a cohort of pediatric patients: clinical, biological and radiological response to treatment with Anakinra

**DOI:** 10.1186/1546-0096-13-S1-P168

**Published:** 2015-09-28

**Authors:** M Pardeo, D Pires Marafon, V Messia, R Nicolai, C Bracaglia, F de Benedetti, A Insalaco

**Affiliations:** 1 Bambino Gesù Children Hospital, Pediatric Medicine-Rheumatology, Rome, Italy

## Introduction

Chronic nonbacterial osteomyelitis (CNO) is the most common autoinflammatory bone disorder in childhood (1). Diagnostic information is provided by TC-99 bone scintigraphy (BS) and/or whole body MRI. Non-steroidal anti-inflammatory drugs (NSAIDs), glucocorticoids, bisphosphonates and tumour necrosis factor inhibitors have been used until now with variable response (2).

## Objectives

To describe clinical, biological and radiological response to treatment with anakinra in patients with CNO refractory to NSAIDs and bisphosphonates.

## Materials and methods

Seven patients (4 females and 3 males) with refractory CNO were treated with anakinra for at least 6 months in our institution. Response to treatment was evaluated assessing clinical manifestations (pain, local swelling, functional impairment), laboratory findings (C-reactive protein (CRP)), erythrocyte sedimentation rate (ESR)) and serum amyloid A level (SAA))) and number of bone lesions on TC-99 BS at the start of treatment and at 6 months.

## Results

The median age at diagnosis and before starting anakinra was 9.7 years (IQR 7.8-14.7) and 13.3 years (IQR 8.0-15.9) respectively. All were treated with NSAIDs and bisphosphonates as first-line therapy. Glucocorticoid therapy was required in one patients with concomitant recurrent fever and pleural effusion. These patients did not respond satisfactorily and anakinra (2 mg/kg/day) was started. At the start of treatment 7/7 patients (100%) had pain, 3/7 (43%) local swelling and 5/7 (71%) functional impairment; at 6 months of follow up 6/7 patients (86%) were completely asymptomatic, with one patient complaining of arthralgia. Before starting anakinra the median CRP, ESR and SAA were 2.7 mg/dl (IQR 1.7-4.9) 26 mm/h (IQR 12-46) and 53 mg/dl (IQR 27-112); at 6 months 5/7 patients (71%) normalized CRP, ESR and SAA; 2/7 had a decrease in inflammatory markers. Before anakinra 59 bone lesions were detected on TC-99 BS. After 6 months of therapy 24/59 lesions (40%) had completely resolved, 1/59 lesions (2%) had partially improved and 29/59 lesions (49%) remained stable. In two patients with persistent high biological inflammatory markers, new lesions (14) developed during treatment.

## Conclusion

Our data suggest that anakinra appears effective in CNO in controlling symptoms and laboratory findings; subclinical bone inflammation was still detectable by BS after 6 months of treatment. Long-term follow-up studies with a larger number of patients are needed.

